# Value of hepatic diffusion-weighted magnetic resonance imaging in evaluating liver fibrosis following transarterial chemoembolization with low doses of chemotherapy

**DOI:** 10.3892/etm.2014.1767

**Published:** 2014-06-06

**Authors:** HONG LI, NA LI, QIN XIANG, YAN ZHOU

**Affiliations:** Department of Radiology, Renhe Hospital, Three Gorges University, Yichang, Hubei 443001, P.R. China

**Keywords:** liver fibrosis, transcatheter arterial chemoembolization, magnetic resonance imaging, diffusion-weighted imaging, apparent diffusion coefficient

## Abstract

The aim of the present study was to investigate the value of apparent diffusion coefficients (ADCs) measured with magnetic resonance (MR) diffusion-weighted imaging (DWI) in evaluating liver fibrosis and curative effects on hepatocellular carcinoma (HCC) following transcatheter arterial chemoembolization (TACE) with low doses of chemotherapy. In total, 84 patients with HCC not recommended for surgical resection underwent TACE. The patients were divided into small dose (n=46) and conventional dose (n=38) chemotherapy groups, and underwent MR-DWI prior to and following TACE. Examination of the four liver fibrosis indexes, hyaluronate, laminin, human procollagen type-III and collagen type-IV, as well as ADC values (b=600 sec/mm^2^), was conducted in the two groups. With small dose chemotherapy, the ADC values were not significantly different preoperatively and postoperatively (P>0.05). By contrast, with a conventional dose, statistically significant differences were observed between the preoperative and postoperative ADC values (P<0.01). ADC values in the small and conventional dose chemotherapy groups prior to the first cycle of TACE were 1.613±0.133×10^−3^ and 1.488±0.248×10^−3^ mm^2^/sec, respectively, while following four cycles of TACE, the ADC values were 1.598±0.147×10^−3^ and 1.206±0.222×10^−3^ mm^2^/sec, respectively. With regard to chemotherapy, the ADC values before and after TACE were significantly different (P<0.05). A significant negative correlation was observed between the ADC values and the fibrosis stage (P<0.05). Therefore, hepatic MR-DWI plays a key role in evaluating liver fibrosis following TACE with low doses of chemotherapy, resulting in improved curative effects of TACE.

## Introduction

Trancatheter arterial chemoembolization (TACE) is one of the preferred methods of treating patients with advanced stage hepatocellular carcinoma (HCC). TACE not only directly kills tumor cells, but also blocks the blood supply to the tumor. However, embolic agents used in chemoembolization, such as iodized oil, may result in liver fibrosis though activating cells around the tumor and excreting numerous types of cytokine. Monitoring the real-time progress of liver fibrosis following TACE and inversing the development has vital clinical significance in prolonging the survival times of patients.

Liver biopsy pathology examinations remain the gold standard in the diagnosis of liver fibrosis (1,2). Due to the uneven distribution of fibrosis tissue, small samples of hepatic tissue in each liver puncture inevitably cause more error. Serum indexes, including hyaluronate (HA), laminin (LN), human procollagen type-III (hPC-III) and collagen type-IV (IV-C), reflect the degree of liver fibrosis. However, the majority of hospitals do not have the equipment required to use these in clinical practice, resulting in limitations in general use (3). Therefore, investigation into a more advanced and reasonable method that can effectively detect the degree of liver fibrosis and is easy to conduct has become an urgent priority.

Magnetic resonance (MR) diffusion-weighted imaging (DWI) reflects the degree of liver fibrosis on a microscopic molecular level and has demonstrated good application prospects (4–6). In the present study, HCC patients were treated with TACE and apparent diffusion coefficient (ADC) values were used to determine the progress of liver fibrosis, with the aim of providing an easy and effective method to detect the progress of liver fibrosis in HCC patients following TACE.

## Materials and methods

### Study objects and grouping

Patients with primary HCC that received TACE between October 2007 and July 2011 were recruited for the study. All the cases were consistent with the diagnosis standard of primary carcinoma of the liver (7). The study selected 84 cases that had normal liver fibrosis indexes prior to treatment, including 38 males and six females with an average age of 49.30±8.84 years. The patients received more than four cycles of TACE and were divided into two groups: Low dose and conventional dose. The low dose group (group A; n=48) were administered low doses of chemotherapeutic agents, including 250 mg 5-fluorouracil (5-FU) and 4 mg mitomycin (MMC), while the conventional group (group B; n=36) received 1,000 mg 5-FU, 8 mg MMC and 40 mg cisplatin. Prior to and at week 4 following the fourth surgery, examinations of the four liver fibrosis indexes (HA, LN, hPC-III and IV-C) and the ADC value were conducted. The study was conducted in accordance with the Declaration of Helsinki and with approval from the Ethics Committee of the Three Gorges University (Yichang, China). Written informed consent was obtained from all the participants.

### TACE procedure

A microcatheter was inserted into the blood-supply artery of the carcinoma to perform embolization with a super-selective intubation technique. Chemotherapeutic drugs, including MMC and 5-FU, were then injected, followed by embolism with hyper liquidness iodinated oil. The dosage of iodinated oil was adjusted according to the size of the focus. Embolism standards included that lesions had good iodinated oil sedimentation, blood supply vessels were predominantly obliterated and the tumor stain had almost vanished. MRI and index analysis determined the next TACE time.

**Figure 1:**
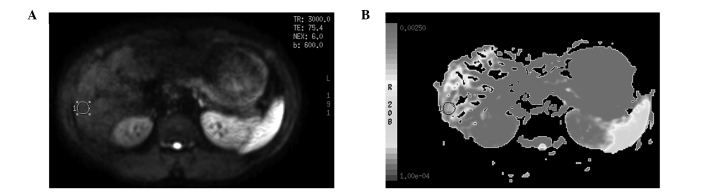
(A) Original DWI following TACE with b=600 sec/mm^2^. (B) Same patient’s pseudo-color graph of peer lever that is the same with picture A. TACE, transcatheter arterial chemoembolization; DWI, diffusion-weighted image.

A Signa HD echospeed 1.5 T MRI system (GE Healthcare, Pittsburgh, PA, USA) with a 16 channel body coil was used for MRI. MR-DWI (1.5T HDe Singa model; American GE Corp, Fairfield, CT, USA) had a b-value of 600 sec/mm^2^ and cross sectional scans. ADC imaging was acquired following computer processing. The center of the porta hepatic plane was selected, as well as points above and below the plane in the right lobe of the liver, to obtain the ADC imaging, from which three regions of interest (ROI) were selected with a size of 50–100 mm^2^. Areas that included the bile ducts, blood vessels, artifacts and the lesion after the treatment of TACE were avoided. The same size ROI was copied and eventually the mean value was calculated as the general ADC value of the right liver lobe (Fig. 1A and B).

### Serology detection

Prior to and at week 4 following the fourth surgery, 5 ml venous blood was collected from all the patients. The samples were placed in test tubes without pyrogen and endotoxin, and were maintained for 2 h at room temperature. Samples were stored in a −20°C refrigerator following centrifugation for 10 min at 6,037 × g. Levels of HA, hPC-III, IV-C and LN were measured using a radioimmunoassay method.

### Statistical analysis

Measurement data are expressed as the mean ± standard deviation. Differences between groups were analyzed by one-factor analysis of variance, while comparisons between groups were performed using the t-test. Pearson’s correlation analysis method was used. All statistical analyses were conducted using SPSS 13.0 statistical software (SPSS, Inc., Chicago, IL, USA), where P<0.05 was considered to indicate a statistically significant difference.

## Results

### ADC values

MR plain scans, DWI and ADC value detection were performed prior to the first TACE cycle and at week 4 following each surgery. ADC values were determined with b=600 sec/mm^2^. Results are shown in Tables I and II.

As shown in Table I, ADC values following each TACE cycle in the low dose group exhibited no statistically significant difference when compared with the preoperative value (P>0.05), while in the conventional dose group, the ADC values decreased markedly and the differences exhibited statistical significance (P<0.05). ADC values of the liver in the two groups following the fourth TACE cycle were significantly lower compared with the value prior to the first TACE cycle (P<0.05), however, group B exhibited a more marked difference (P<0.01; Table II).

### Liver fibrosis indexes

Levels of the four serum liver fibrosis indexes (HA, hPC-III, IV-C and LN) were measured prior to and following each TACE cycle (Table III).

Data of the liver fibrosis indexes shown in Tables II and III were analyzed with a t-test. The results demonstrated that the value of each liver fibrosis index in group A had no significant difference following each TACE cycle when compared with the preoperative value. In group B, following the first TACE cycle, the same results were observed as group A, however, following the second, third and fourth TACE cycle, each liver fibrosis index was observed to increase (P<0.05), with HA exhibiting the most significant change (P<0.01).

### Correlation analysis between liver fibrosis and ADC values

**Figure 2:**
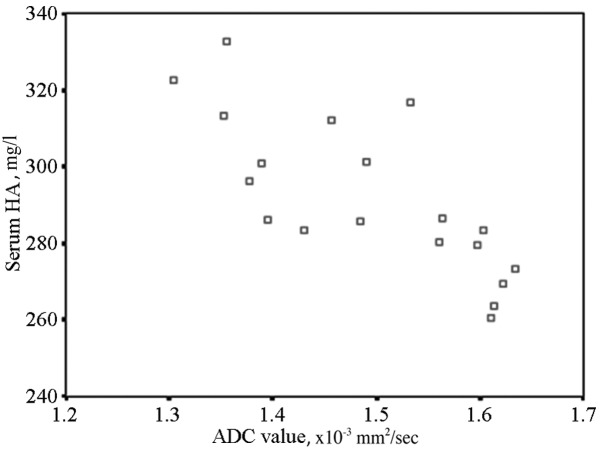
Scatter diagram showing the correlation between the level of HA and ADC values. HA, hyaluronate; ADC, apparent diffusion coefficient.

Liver fibrosis progress exhibited significant effects on the serum liver fibrosis indexes. HA was the most significant of the four indicators, thus, HA should be used to evaluate the degree of liver fibrosis. When comparing HA with the ADC value at the same time period using Pearson’s method, the two indicators exhibited a significant negative correlation (r=0.535, P<0.01). The results are shown in the scatter diagram (Fig. 2).

## Discussion

In the development of liver fibrosis, the main pathological change is the aggregation of a large number of collagen fibers in the extracellular matrix. The deposition of collagen fibers reduces extracellular clearance, which further reduces the moisture capacity, as collagen fiber itself does not have a high moisture capacity. This limits the diffusion movement of water molecules, thus, resulting in a decrease in ADC values (8–10). The more deposition of collagen fibers in the extracellular matrix, the more progressive the liver fibrosis and the more significant the decrease in ADC values (2). Lewin *et al* (11) performed MR-DWI in 54 patients, with the results also showing that ADC values were correlated to liver fibrosis grading (3,12). Previous studies have also demonstrated that the liver and spleen ADC ratio may reflect the progression of liver fibrosis more specifically. The study by Sun *et al* (13) included 41 patients with chronic liver disease, in which MR-DWI and blood biochemical index analysis was performed. The study indicated that a logistic regression model constructed with ADC values and multiple blood biochemical indexes had even higher diagnostic accuracy compared with single detection. All these studies hypothesized that ADC values aided the diagnosis, staging and prediction of liver fibrosis. In the present study, lower and conventional doses of chemotherapy in TACE were compared, and the ADC values of the conventional group exhibited statistical significance following each TACE cycle when compared with the preoperative values. Therefore, to a certain extent, we hypothesized that ADC values can replace liver fibrosis indexes in the evaluation of liver fibrosis progression.

A number of studies have indicated that DWI, as an invasive MR functional imaging technique, may eliminate the T2 penetration effect, and reflect the pacing of hydrone diffusion more accurately and provide quantitative indicators. DWI may be used to reflect the characteristics of the organization and curative effects from a molecular level, and has demonstrated a superior value in the evaluation of interventional therapeutics in HCC (14,15).

In TACE treatment of HCC, chemotherapeutic drugs and iodized oil may activate cells around the tumor, causing the excretion of numerous types of cytokine and eventually leading to liver fibrosis. The prevention or delay of liver fibrosis, as well as a scientific method for the monitoring and assessment of liver fibrosis, are becoming increasingly studied for the interventional therapy of HCC. Due to the cytotoxic effect of large doses of chemotherapeutic drugs, tumor cells undergo necrosis or apoptosis and a large number of collagen fibers are deposited in the extracellular matrix of normal liver cells, resulting in a marked decrease in the ADC value compared with the preoperative value. ADC values were analyzed in non-tumor areas of the liver in the conventional group and no significant difference was observed between the postoperative and preoperative values following the first TACE cycle. ADC values decreased following the second and third surgeries (P<0.05), and after the fourth TACE cycle, the ADC values had decreased significantly (P<0.01). The results confirmed that repeated cycles of TACE with large doses of chemotherapeutic drugs can aggravate the process of liver fibrosis.

Kamada *et al* (16) indicated that hepatic cancer cells were not sensitive to chemotherapeutic drugs, thus, reducing the dose of chemotherapeutic agents had little effect on the recent and forward curative effects. The present study analyzed ADC values in non-tumor areas of the liver in low dose groups and no significant difference was observed between the postoperative and preoperative values following each TACE cycle (P>0.05). However, the ADC values significantly decreased following the fourth surgery, as compared with the value prior to the first TACE (P<0.05). Thus, it is clear that ADC values exhibit no marked decrease following surgery as a result of reducing the dose of chemotherapeutic drugs. This observation further indicated that liver fibrosis had no or little progression, thus, low doses of chemotherapeutic drugs in TACE may improve the curative effect.

Correlation analysis was performed between the liver fibrosis indexes and ADC values. Due to the limitations of liver pathological examinations in the evaluation of liver fibrosis (17,18), the use of four blood serum indexes of liver fibrosis is generally accepted. The liver tissue proliferation weakened, of which the indices level of liver fibrosis increased as well as the serological index. Extracellular matrix abnormal hyperplasia and deposition lead to an increase in blood metabolites, which further induces the levels of serum liver fibrosis indexes to increase. By contrast, the over-deposition of extracellular matrix aggravates the barricade of the extracellular space and limits the diffusing capacity of hydrone, causing the ADC values to decrease. Previous studies (19,20) have indicated that there is a correlation between ADC values and serum HA levels. The present study identified a varying extent of correlation between ADC values of liver non-tumor areas and serum indicators, including HA, PCIII, C-IV and LN. In the conventional group, the liver fibrosis indexes increased following the second, third and fourth cycle of TACE (P<0.05), with HA increasing most significantly (P<0.01). While in the low dose group, a significant negative correlation was observed between HA levels and ADC values, as analyzed with Pearson’s method (r=−0.535, P<0.01); the result exhibited statistical significance.

In conclusion, the ADC value can not only be used to evaluate the extent of liver fibrosis progression, but also with its unconspicuous decline to indicate that it had slower or no progress to liver fibrosis in patients with lower doses of chemotherapeutics, and thus to improve the curative effect of TACE.

## Figures and Tables

**Table I:** ADC values prior to and following four cycles of TACE in groups A and B.

	Group A (n=48)			Group B (n=36)		
				
TACE cycle	Preoperative (x10^−3^ mm^2^/sec)	Postoperative (x10^−3^ mm^2^/sec)	P-value	Preoperative (x10^−3^ mm^2^/sec)	Postoperative (x10^−3^ mm^2^/sec)	P-value
First	1.613±0.133	1.595±0.124	0.2531	1.598±0.147	1.433±0.151	0.0435
Second	1.595±0.124	1.564±0.165	0.1574	1.433±0.151	1.359±0.159	0.0371
Third	1.564±0.165	1.526±0.213	0.1128	1.359±0.159	1.286±0.123	0.0395
Fourth	1.526±0.213	1.488±0.248	0.0981	1.286±0.123	1.206±0.222	0.0366

ADC, apparent diffusion coefficient; TACE, transcatheter arterial chemoembolization.

**Table II:** ADC values prior to the first cycle of TACE and following the fourth TACE cycle in groups A and B.

Group	Prior to the first TACE (x10^−3^ mm^2^/sec)	Following the fourth TACE (x10^−3^ mm^2^/sec)	P-value
A (n=48)	1.613±0.133	1.488±0.248	0.0342
B (n=36)	1.598±0.151	1.206±0.222	0.0057

The preoperative and postoperative values being compared. ADC, apparent diffusion coefficient; TACE, transcatheter arterial chemoembolization.

**Table III:** Liver fibrosis indexes prior to and following TACE cycles.

	Group A (n=48)		Group B (n=36)	
		
Fibrosis indexes	Preoperative	Postoperative	Preoperative	Postoperative
First TACE				
HA (mg/l)	259.81±78.16	263.81±87.24	263.81±87.24	279.47±95.39
PC-III (μg/l)	182.11±65.27	186.86±58.05	180.11±65.27	199.16±83.25
IV-C (μg/l)	121.20±43.46	127.20±38.40	119.20±43.46	124.13±25.43
LN (μg/ml)	159.03±33.66	166.51±38.19	157.03±32.55	169.08±31.25
Second TACE				
HA (mg/l)	276.67±81.23	273.24±95.57	279.47±95.39	316.15±124.48^b^
PC-III (μg/l)	189.11±78.82	193.23±88.07	182.11±81.34	243.16±89.52^a^
IV-C (μg/l)	123.27±50.35	123.27±50.35	127.27±50.35	179.71±69.89^a^
LN (μg/ml)	177.86±25.53	177.86±25.53	164.86±25.53	192.21±29.78^a^
Third TACE				
HA (mg/l)	276.63±91.14	296.63±102.16	286.63±102.16	332.45±128.63^b^
PC-III (μg/l)	190.66±85.15	203.81±85.25	210.46±75.15	236.16±88.46^a^
IV-C (μg/l)	130.00±55.37	139.45±60.66	150.20±57.35	188.16±70.13^a^
LN (μg/ml)	183.86±30.75	183.86±30.75	183.86±30.75	201.58±68.43^a^
Fourth TACE				
HA (mg/l)	293.63±98.05	296.07±101.11	311.63±103.21	352.11±132.36^b^
PC-III (μg/l)	198.57±85.11	211.66±81.74	218.46±75.15	255.16±78.21^a^
IV-C (μg/l)	140.08±62.22	148.33±61.84	159.20±57.35	199.16±72.13^a^
LN (μg/ml)	190.10±35.77	197.45±36.88	189.86±35.77	231.58±78.45^a^

a P<0.05;

b P<0.01.

TACE, transcatheter arterial chemoembolization.
